# Transformed lymphoplasmacytic lymphoma involving the main carina: A case report

**DOI:** 10.3892/ol.2013.1369

**Published:** 2013-05-29

**Authors:** MAKOTO NAKAO, TETSUYA OGURI, MIKINORI MIYAZAKI, HISATOSHI HIJIKATA, MIDORI YOKOYAMA, EIJI KUNII, TAKEHIRO UEMURA, OSAMU TAKAKUWA, HIROTSUGU OHKUBO, KEN MAENO, AKIO NIIMI

**Affiliations:** Department of Medical Oncology and Immunology, Nagoya City University, School of Medical Sciences, Nagoya, Aichi 467-8601, Japan

**Keywords:** endobronchial tumor, lymphoplasmacytic lymphoma, transformation, autofluorescence bronchoscopy

## Abstract

A 41-year-old male was admitted to Nagoya City University Hospital subsequent to experiencing a cough with bloody sputum for a few days. The patient had a 4-year history of lymphoplasmacytic lymphoma (LPL) and had achieved a good partial response to anticancer chemotherapy. Chest computed tomography (CT) showed an endobronchial tumor of the main carina. A bronchoscopy revealed an exophytic tumor at the main carina, and autofluorescence imaging bronchovideoscopy showed that the tumor and surrounding area were magenta in color. The biopsy specimens demonstrated that the endobronchial tumor was composed of large atypical lymphoid cells. The patient was diagnosed with a high-grade transformation of LPL. In addition to describing a rare case of transformed LPL involving the main carina, the present study also summarizes and discusses endobronchial lymphomas, with a brief review of a number of published studies.

## Introduction

Malignant lymphomas (ML) frequently involve intrathoracic organs, but endobronchial involvement (EBI) is relatively rare (1–3). The present study describes the case of a patient with lymphoplasmacytic lymphoma (LPL) transforming to large cell lymphoma, characterized by an endobronchial tumor of the main carina. To the best of our knowledge, this type of progression of LPL has not previously been reported. Notably, the present study used an autofluorescence imaging (AFI) bronchovideoscope system to evaluate the invasion of ML into the surrounding mucosa. The study provides further insight into the clinical significance of the EBI of ML and the utility of bronchoscopic examination in these patients. Written informed consent was obtained from the patient.

## Case report

A 41-year-old male was referred to the Respiratory Division of Nagoya City University Hospital subsequent to experiencing a cough with bloody sputum for a few days. The patient had a 4-year history of a low-grade non-Hodgkin’s lymphoma (NHL) with IgM paraproteinemia, diagnosed as LPL. The primary LPL lesion was in the bone marrow, with no lesions detected on computed tomography (CT) or 18F-fluorodeoxyglucose-positron emission tomography (FDG-PET). The patient had been treated with chemotherapy, including 8 courses of R-COP therapy (rituximab, cyclophosphamide, oncovin and prednisolone), 12 courses of fludarabine and 5 courses of bendamustine, and had attained a good partial response (PR). The cough with bloody sputum was first noticed ∼1 month after the final dose of chemotherapy.

**Figure 1.**
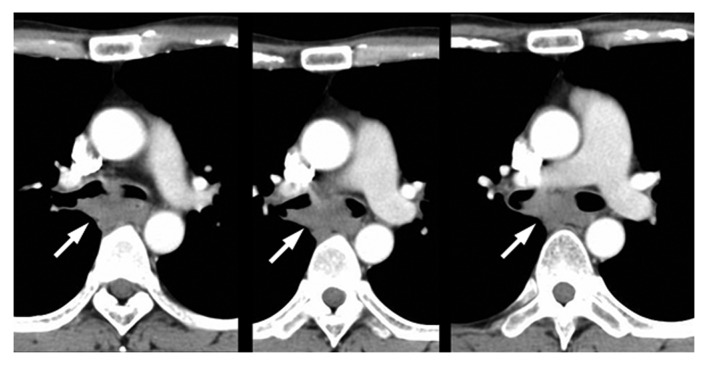
Contrast-enhanced computed tomography (CT) scan on admission showing subcarinal lymphadenopathy and an endobronchial tumor at the main carina.

**Figure 2.**
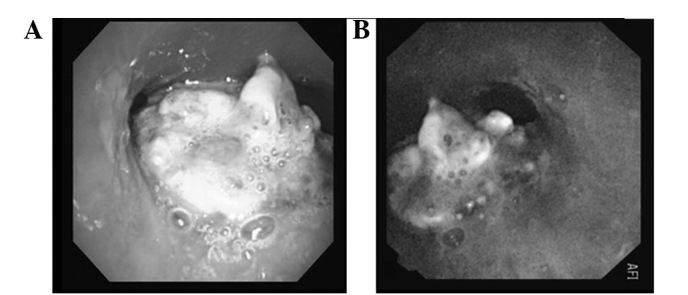
(A) Bronchoscopic photograph showing an exophytic tumor with a rough surface and a white moss-like appearance at the main carina. (B) Bronchovideoscope system autofluorescence imaging (AFI) revealed an exphytic tumor.

**Figure 3.**
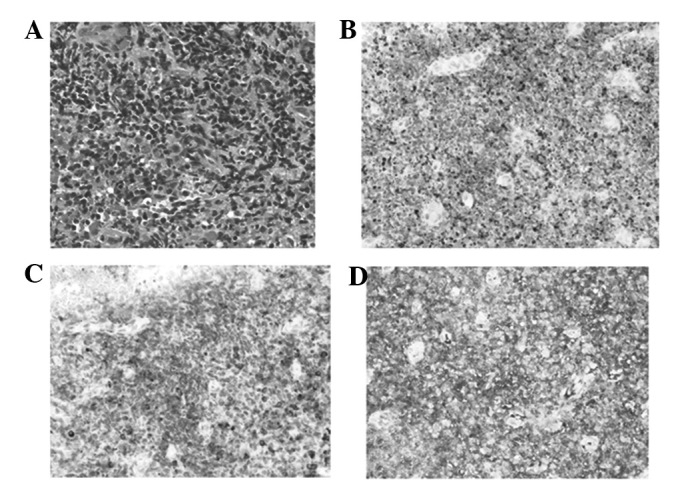
(A) Biopsy specimen from the endobronchial lesion (hematoxylin and eosin staining). (B) Immunohistochemical staining with anti-CD20 is positive. (C) Immunohistochemical staining with anti-CD79a is positive. (D) Immunohistochemical staining with anti-κ is positive (Magnification, ×400). In immunohistochemical staining, the darker areas represent positive staining.

A physical examination revealed crackles in each lung and a oxyhemoglobin saturation of 96% in normal room air. A CT scan showed subcarinal lymphadenopathy and an endobronchial tumor involving the main carina (Fig. 1). The patient’s platelet count was low [63,000/*μ*l (normal, >148,000/*μ*l)], and the concentration of circulating interleukin-2 receptor (IL-2R) was slightly elevated [538 U/ml (normal, <519 U/ml)]. The lactate dehydrogenase (LDH) level was within the normal range. The IgG, IgA and IgM concentrations were 408 mg/dl (normal, 870–1700 mg/dl), 11 mg/dl (normal, 110–410 mg/dl) and 187 mg/dl (normal, 35–220 mg/dl), respectively. A bronchoscopic examination showed an exophytic tumor with rough surfaces and a white, moss-like appearance at the main carina (Fig. 2). An autofluorescence imaging (AFI) examination showed that the exophytic tumor and surrounding area were magenta in color. A transbronchial biopsy (TBB) specimen showed large atypical cells (Fig. 3). Immunohistochemical staining was positive for CD20 and CD79a and negative for CD3 and CD10. The cells were weakly positive for IgM and the κ/λ ratio was high. The patient was diagnosed with a high-grade transformation of LPL involving the main carina.

The patient was treated with 2 courses of DeVIC therapy (dexamethasone, etoposide, ifosfamide and carboplatin), and achieved a complete response (CR), as confirmed by FDG-PET. Thereafter, the patient underwent an allogeneic stem cell transplantation and is currently (15 months post-treatment) alive without recurrence.

## Discussion

The present study describes a rare case of transformed LPL with carinal involvement. Although ML frequently involves the intrathoracic organs, endobronchial involvement (EBI) in NHL is rare, even in patients with advanced disease (3,4). To the best of our knowledge, a high-grade transformation of LPL detected as an endobronchial tumor has not previously been reported.

LPL is classified as a low-grade lymphoma and constitutes <5% of all NHLs. LPL occurs in older adults, usually involving the bone marrow, lymph nodes and spleen, while extranodal involvement and a leukemic phase are rare (5). LPL tumors consist of a diffuse arrangement of small B lymphocytes with variable degrees of plasmacytoid differentiation. The clinical presentation usually consists of a disseminated disease, with >20% of patients having monoclonal IgM paraproteinemia and hyperviscosity symptoms (5). The median survival of patients with LPL is 50–60 months due to the transformation to large cell lymphoma. The primary LPL lesion in the present patient was in the bone marrow, and IgM paraproteinemia was detected. The transformation to large cell lymphoma occurred ∼48 months after the initial diagnosis of LPL. With the exception of the age of onset of the LPL and EBI, the patient had a typical clinical course.

Endobronchial lymphomas have been classified into 2 types; diffuse submucosal infiltrations in the presence of intra- and extra-thoracic lymphoma (type I), and central airway involvement due to a solitary mass in the absence of clinically-apparent systemic lymphoma (type II) (6,7). In Japan, however, endobronchial lymphomas have been classified into 3 types, characterized by a raised tumor mass (type I), multiple submucosal nodules (type II) and diffuse submucosal infiltration (type III) (2). The present patient may be classified endoscopically as type II according to the former classification system and as type I according to the latter. Although the more common type of EBI accompanying NHL differs in various studies (2,6,7), this may be due to the varying characteristics of NHL.

Five mechanisms of endobronchial metastasis have been proposed: i) direct bronchial invasion of a parenchymal mass; ii) direct bronchial invasion of a mediastinal mass; iii) lymphatic spread to peribronchial connective tissues; iv) transbronchial aspiration of tumor emboli; and v) direct hematogenous metastasis (8). The most common mechanisms regarding endobronchial NHLs are mechanisms ii) and iii) (8). Chest CT scans in the present patient showed that the endobronchial tumor was from mediastinal lymphadenopathy, suggestive of mechanism ii).

The majority of EBIs occur in patients with systemic or relapsed/refractory disease, and are more frequent in patients with Hodgkin’s disease than in those with NHL (2,7). Although the most frequent EBI loci observed in NHL patients differ, the main and lobar bronchi are the most common sites (2,4,7). The most common histological subtypes of endobronchial lymphoma are considered to be bronchus-associated lymphoid tissue lymphoma and diffuse B-cell lymphoma (6,7,9), although too few patients have been assessed to draw definitive conclusions. Further investigations of EBI accompanying NHL are warranted.

Autofluorescence bronchoscopy is an important tool in the early detection of preinvasive bronchial lesions, including squamous dysplasia, carcinoma *in situ* and early hilar lung carcinoma (10). A new type of autofluorescence bronchoscopy, AFI, has been revealed to more precisely distinguish preinvasive bronchial lesions and bronchitis (10). The AFI examination of the present patient demonstrated that the exophytic tumor and its surrounding areas were magenta in color. Since the mucosal surface surrounding the tumor was endoscopically smooth, submucosal invasion of the lymphoma was suspected. However, as only a few studies to date have described the use of AFI in patients with endobronchial lymphoma (11,12), further studies are necessary to assess the usefulness of AFI in ML patients with EBI.

In conclusion, the present study describes a rare case of LPL involving the main carina. Bronchoscopic examinations with TBB and AFI led to a diagnosis of a transformation to large cell lymphoma. These findings may provide further insight into endobronchial lymphoma and, particularly in patients with refractory low-grade lymphoma, emphasize the importance of histological examination of newly developed lesions.
